# Osteopontin-Enhanced Autophagy Attenuates Early Brain Injury via FAK–ERK Pathway and Improves Long-Term Outcome after Subarachnoid Hemorrhage in Rats

**DOI:** 10.3390/cells8090980

**Published:** 2019-08-27

**Authors:** Chengmei Sun, Budbazar Enkhjargal, Cesar Reis, Tongyu Zhang, Qiquan Zhu, Keren Zhou, Zhiyi Xie, Lingyun Wu, Jiping Tang, Xiaodan Jiang, John H. Zhang

**Affiliations:** 1 The National Key Clinical Specialty, The Engineering Technology Research Center of Education Ministry of China, Guangdong Provincial Key Laboratory on Brain Function Repair and Regeneration, Department of Neurosurgery, Zhujiang Hospital, Southern Medical University, 253 Gongye Road, Guangzhou 510282, China; 2 Department of Physiology and Pharmacology, Loma Linda University, 11041 Campus St, CA 92354, USA; 3 Key Laboratory of Mental Health of the Ministry of Education, Guangdong-Hong Kong-Macao Greater Bay Area Center for Brain Science and Brain-Inspired Intelligence, Southern Medical University, 1023 South Shatai Road, Guangzhou 510515, China

**Keywords:** osteopontin, subarachnoid hemorrhage, autophagy, hippocampal injury, delayed brain injury

## Abstract

Osteopontin (OPN) enhances autophagy, reduces apoptosis, and attenuates early brain injury (EBI) after a subarachnoid hemorrhage (SAH). A total of 87 Sprague–Dawley rats were subjected to sham or SAH operations to further investigate the signaling pathway involved in osteopontin-enhanced autophagy during EBI, and the potential effect of recombinant OPN (rOPN) administration to improve long-term outcomes after SAH. Rats were randomly divided into five groups: Sham, SAH + Vehicle (PBS, phosphate-buffered saline), SAH + rOPN (5 μg/rat recombinant OPN), SAH + rOPN + Fib-14 (30 mg/kg of focal adhesion kinase (FAK) inhibitor-14), and SAH + rOPN + DMSO (dimethyl sulfoxide). Short-term and long-term neurobehavior tests were performed, followed by a collection of brain samples for assessment of autophagy markers in neurons, pathway proteins expression, and delayed hippocampal injury. Western blot, double immunofluorescence staining, Nissl staining, and Fluoro-Jade C staining assay were used. Results showed that rOPN administration increased autophagy in neurons and improved neurobehavior in a rat model of SAH. With the administration of FAK inhibitor-14 (Fib-14), neurobehavioral improvement and autophagy enhancement induced by rOPN were abolished, and there were consistent changes in the phosphorylation level of ERK1/2. In addition, early administration of rOPN in rat SAH models improved long-term neurobehavior results, possibly by alleviating hippocampal injury. These results suggest that FAK–ERK signaling may be involved in OPN-enhanced autophagy in the EBI phase after SAH. Early administration of rOPN may be a preventive and therapeutic strategy against delayed brain injury after SAH.

## 1. Introduction

Aneurysmal subarachnoid hemorrhage (SAH) is a devastating subtype of hemorrhagic stroke with high mortality and disability [1,2]. Early brain injury (EBI), which occurs within 72 h after SAH is the primary cause of the poor outcomes for the high mortality and delayed neurological deficits in patients who suffer from subarachnoid hemorrhage (SAH) [3,4].

In the past few decades, researchers have implicated many pathological mechanisms contributing to EBI. The rupture of intracranial aneurysms initially lead to emergency illness such as elevated intracranial pressure, decreased cerebral blood flow, and decreased cerebral perfusion pressure. These events consecutively initiate various cascades of pathophysiological processes such as inflammation, oxidative stress, blood–brain barrier dysfunction, and apoptosis [5,6]. Among all these processes, neuronal apoptosis is an important pathologic event in the pathogenesis of SAH-induced EBI [7].

Autophagy is an intracellular process that maintains cellular homeostasis and recycles damaged organelles and proteins [8]. An elevated autophagy level and activated autophagy-related pathway proteins have been demonstrated in experimental SAH [9,10]. In summary of these previous studies, autophagy is a pro-survival mechanism, attempting to inhibit neuronal apoptosis in EBI after SAH [9,11,12,13,14].

Osteopontin (OPN) is a pleiotropic glycoprotein, widely expressed in bone, immune cells, smooth muscle, epithelial and endothelial cells, neurons, adipocytes, Kupffer cells, etc. [15]. OPN plays a crucial role in the regulation of apoptosis, angiogenesis, and in cancer progression [16,17]. Previous studies also suggested neuroprotective effects of OPN after SAH in rats [7,18]. However, there has been no previous research about the role of OPN after SAH in human studies. 

Since studies showed that OPN was a potential activator of autophagy in the in vitro model of abdominal aorta aneurysm [19] and hepatocellular carcinoma [20], one of our recent studies investigated the effect of recombinant OPN (rOPN) administration on autophagy after SAH in rat endovascular perforation models. We found increased expressions of endogenous OPN and autophagy-related proteins during the EBI phase. After rOPN administration, there were elevated autophagy protein expressions and a decreased apoptosis level in the perforated side of rat brains 24 h after SAH. We also observed an interaction between autophagy initiator Beclin 1 and apoptosis-related protein Caspase-3 which could be regulated by rOPN administration at 24 h after SAH [21]. However, the molecular mechanism involved in OPN-enhanced autophagy after SAH has not been studied yet. Moreover, there has been no previous research about whether rOPN administration can influence long-term outcome after SAH.

One previous study suggested that OPN could activate autophagy through both of its receptors, integrin and CD44 [19]. Focal adhesion kinase (FAK) is one of the major integrin signaling mediators and is activated via autophosphorylation on Y397 [22]. One of our previous studies already showed that OPN’s anti-apoptosis effect after SAH is through FAK signaling [7]. Thus, to better understand the underlying mechanisms of OPN-enhanced autophagy after SAH, we designed the mechanism part of the current study using the specific inhibitor of FAK. Moreover, we investigated whether rOPN-attenuated EBI has a beneficial effect on long-term outcomes after experimental SAH.

## 2. Materials and Methods

### 2.1. Animals

A total of 87 adult male Sprague–Dawley rats (290–330 g, Harlan, Indianapolis, IN, USA) were housed in a room with constant temperature (25 °C), humidity control, 12 h/12 h light/dark cycle and with free access to food and water. All the experimental procedures were approved by the Institutional Animal Care and Use Committee (IACUC) of Loma Linda University (No. 8170018), and in accordance with the National Institutes of Health’s Guide for the Care and Use of Laboratory Animals. All experiments were reported with the ARRIVE (Animal Research: Reporting In Vivo Experiments) guidelines for reporting in vivo experiments.

### 2.2. SAH Model

The SAH endovascular perforation model was conducted as previously described [23,24]. Briefly, rats were intubated and maintained with 3% isoflurane in 70%/30% medical air/oxygen by a rodent ventilator (Harvard Apparatus, Holliston, MA, USA). Rats were placed in a supine position, and the neck was opened with a sharp scalpel in the midline. Subsequently, we exposed the left carotid artery and its branches. A sharpened 4–0 nylon suture was inserted into the left internal carotid artery through the external carotid artery stump until resistance was detected. The suture was further advanced 3 mm to perforate the bifurcation of the anterior and middle cerebral artery, followed by immediate withdrawal. Rats in the Sham group underwent the same procedure; however, the suture was withdrawn without puncture. After removal of the suture, the skin incision was closed and the rats were placed on a heating pad to maintain the body temperature. Respiration and mucosal color were monitored every 10 min until animals could maintain an upright posture and walk normally. Then they were transferred back to their home cage and re-evaluated at the end of the day [25].

### 2.3. SAH Grading

The assessment of SAH grading score was performed as previously described at 24 h after SAH by an independent observer blinded to the experimental group information. The basal cistern was divided into six segments, each graded from 0 to 3, depending on the amount of blood clots. The total score was calculated by adding all area scores (maximum SAH grade = 18). Animals with mild SAH (SAH grade ≤ 8) were excluded from the current study as previously described [1,4].

### 2.4. Experimental Design

**Figure 1:**
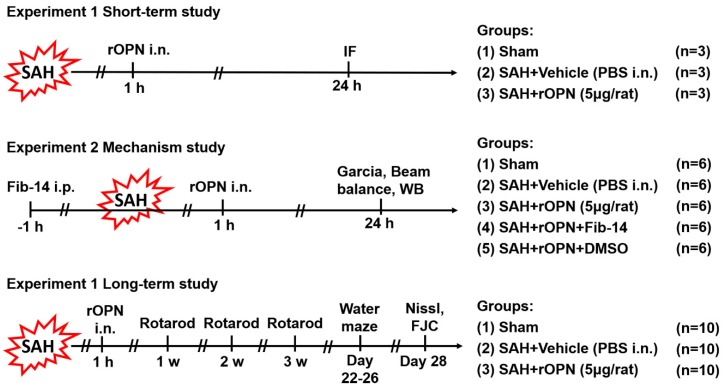
Experimental design. SAH, subarachnoid hemorrhage; WB, western blot; PBS, phosphate-buffered saline; IF, immunofluorescence; rOPN, recombinant osteopontin; i.*n*., intra nasal administration; Fib-14, focal adhesion kinase (FAK) inhibitor 14; i.p., intraperitoneal administration; DMSO, dimethyl sulfoxide; FJC, Fluoro-Jade C.

Experimantal design of the current study is described below and illustrated in Figure 1.

#### 2.4.1. Experiment 1

To determine the effect of rOPN on autophagy protein expression in neurons after SAH, nine rats were randomly divided into three groups (*n* = 3 per group) for double immunofluorescence staining and subsequent double positive-stained cell counting: Sham, SAH + Vehicle (30 μL PBS), SAH + rOPN (5 μg/rat recombinant osteopontin in 30 μL PBS, 6359-OP-050, R&D Systems, Minneapolis, MN, USA). Vehicle or rOPN was delivered via the nasal route 1 h after SAH induction, and brain samples were collected 24 h after SAH. The intranasal rOPN dosage was selected based on previous studies [7,21].

#### 2.4.2. Experiment 2

To investigate the signaling pathway involved in rOPN-enhanced autophagy after SAH, 30 rats were randomly assigned to five groups (*n* = 6 per group): Sham, SAH + Vehicle (30 μL PBS), SAH + rOPN (5 μg/rat recombinant osteopontin in 30 μL PBS), SAH + rOPN + Fib-14 (30 mg/kg in 200 μL of 25% DMSO in PBS) and SAH + rOPN + DMSO (200 μL of 25% DMSO in PBS) for western blot analysis. FAK inhibitor 14 (Fib-14, ab146739, Abcam, Cambridge, MA, USA) and DMSO were intraperitoneally (i.p.) delivered 1 h before SAH induction. Vehicle or rOPN was delivered via the nasal route 1 h after SAH induction. The intraperitoneal Fib-14 dosage was selected based on a previous study [26], and according to the manufacturer’s instructions ab146739 is a selective focal adhesion kinase (FAK) autophosphorylation inhibitor, with no significant activity at other kinases including EGFR, PDGFR, and IGF-RI. Neurological tests including modified Garcia test and beam balance test were evaluated 24 h after SAH, after which brain samples were collected for western blot analysis.

#### 2.4.3. Experiment 3

To evaluate the long-term effect of rOPN after SAH, 30 rats were randomly divided into three groups (*n* = 10 per group): Sham, SAH + Vehicle (30 μL PBS), SAH + rOPN (5 μg/rat recombinant osteopontin in 30 μL PBS) for evaluation of long-term neurological scores and histological results including Nissl staining and Fluoro-Jade C staining. Vehicle or rOPN was delivered via the nasal route 1 h after SAH induction. Neurobehavior tests were carried out from day 7 to day 26 after SAH. Brain samples were collected on the 28th day after SAH.

### 2.5. Intranasal Administration 

Vehicle or rOPN was delivered via the intranasal route 1 h after SAH induction as previously described [21]. Rats under 2% isoflurane anesthesia were placed in a supine position and a total volume of 30 μL PBS or rOPN was administered alternately into the left and right nares, 5 μL each time with an interval of 2 min between each administration.

### 2.6. Short-Term Neurobehavior Assessment

Short-term neurobehavior tests, including modified Garcia test and beam balance test, were performed by an independent investigator blinded to the experimental group information at 24 h after SAH as previously described [27]. The modified Garcia test (maximum score = 18) includes judgments of spontaneous activity, spontaneous movement of all limbs, forelimbs outstretching, response to vibrissae touch, body proprioception, and climbing capacity.

The beam balance test was conducted to assess the ability of rats to walk on a 15-mm-wide wooden beam for 1 min [1]. The mean score was calculated based on three consecutive trials scored from 0 to 4 according to the walking ability. Higher scores indicated better neurological function.

### 2.7. Long-Term Neurobehavior Assessment 

The rotarod test and Morris water maze were performed to evaluate the long-term neurobehavioral function of animals by an independent investigator blinded to the grouping information.

The rotarod test was performed by the end of the first, second, and third-week post-SAH to assess sensorimotor coordination and balance as previously described [28]. The rotarod apparatus (Columbus Instruments, Columbus, OH, USA) consists of a horizontal rotating cylinder (diameter = 7 cm) which is divided into four 9.5-cm-wide lanes. After animals were stably placed on the cylinder, its rotating speed started at 5 revolutions per minute (RPM) and 10 RPM, respectively, and accelerated by 2 RPM every 5 s. Latency to fall off the cylinder was recorded by a photo beam circuit.

The Morris water maze test was performed from day 22 to day 26 after SAH to evaluate spatial learning capacity as previously described [29]. Briefly, a platform was placed at the center of one of the quadrants. Rats were placed using a semi-random set of start locations to find a visible platform above the water level in 60 s. Then rats were guided to the platform and stayed on it for 5 s. On the last day, the animals were tested with a 60-s probe trial with the platform submerged in the water. Swimming velocity, swimming distance, and escape latency were recorded by a computerized tracking system (Video Tracking System SMART-2000 (San Diego Instruments Inc., CA, USA)). Then the platform was removed from the water, and probe quadrant duration was also recorded by the same video recording system.

### 2.8. Western Blot Analysis 

Western blot tests were performed as previously reported [30]. Briefly, whole left hemispheres of rats in different groups were isolated and collected 24 h after SAH. After protein extraction, equal amounts of protein samples were loaded onto each lane of SDS-PAGE gel. After electrophoresis, the protein samples were transferred onto a nitrocellulose membrane for blocking and incubation at 4 °C overnight with the following primary antibodies: Anti-osteopontin (1:1000, sc-21742), anti-ERK 1/2 (phosphor-Thr202/Tyr204) (1:200, sc-16982), anti-ERK1/2 (1:2000, sc- 514302), and anti-β-actin (1:5000, I-19) from Santa Cruz Biotechnology Inc., TX, USA; anti-Beclin 1 (1:1000, NB500-249), anti-ATG 5 (1:500, NB110-53818), anti-LC3 (1:5000, NB600-1384) from Novus Biologicals, CO, USA; anti-FAK (phospho-Y397) (1:1000, ab81298), anti-FAK (1:1000, ab131435), anti-SQSTM1/p62 (1:5000, ab56416) from Abcam, Cambridge, MA, USA). Secondary antibodies used were anti-mouse (1:5000, sc-2031), anti-goat (1:5000, sc-2354) from Santa Cruz Biotechnology Inc., and anti-rabbit (1:5000, 2839792) from EMD Millipore Corporation, MA, USA.

### 2.9. Double Immunofluorescence Staining 

Double immunofluorescence staining was performed as previously described [31]. A series of 8-μm-thick frozen brain tissue slices were prepared. The primary antibodies used were anti-NeuN (1:500, ab177487) from Abcam, Cambridge, MA, USA; anti-osteopontin (1:100, sc-21742) from Santa Cruz Biotechnology Inc., TX, USA; anti-Beclin 1 (1:100, NB500-249) and anti-LC3 (1:5000, NB600-1384) from Novus Biologicals, CO, USA. Corresponding secondary antibodies used were purchased from Jackson ImmunoResearch, West Grove, PA, USA, and applied at the concentration of 1:500. After staining, the sections were visualized and photographed with a fluorescence microscope (Leica Microsystems, Germany) at ×400 magnification by an independent observer. Three rat brains per group with six sections per brain were used for quantification analysis. Image J software (Image J 1.4, NIH, Bethesda, MD, USA) was used for cell counting. The data were presented as the average number of double-labeled cells per square millimeter (cell/mm^2^).

### 2.10. Nissl Staining 

On the 28th day after SAH, the animals were sacrificed after deep anesthesia. They were transcardially perfused with 300 mL of ice-cold PBS, followed by 300 mL of formalin solution. Hippocampal coronal sections 16 μm thick were taken every 500 μm at −3 to −4.3 mm from the bregma according to the coordinates in “Paxinos and Watson method” as previously described [29] using a cryostat (CM3050S; Leica Microsystems, Buffalo Grove, IL, USA). Nissl staining was performed using 0.5% crystal violet as previously described [32]. The sections were observed under a light microscope at 200× magnification by an independent observer for morphologic signs of hippocampal injury and neuronal degeneration (neuronal shrinkage, hyperchromasia, and vacuolization).

### 2.11. Fluoro-Jade C Staining 

Fluoro-Jade C (FJC) staining was performed to detect degenerating neurons with a modified FJC ready-to-dilute staining kit (Biosensis, Thebarton, South Australia) according to the manufacturer’s instructions [33]. Samples were collected on day 28 after SAH. Hippocampal coronal sections 16 μm thick were taken every 500 μm at −3 to −4.3 mm from the bregma in the same manner as in Nissl staining. Three rat brains per group (six hippocampal sections per brain) were used for quantification analysis in the hippocampal area. Images of FJC-positive neurons in designated locations were captured at 200× magnification by an independent observer. Image J software (Image J 1.4, NIH, USA) was used for cell counting. The data were presented as the average number of FJC-positive neurons per square millimeter (cell/mm^2^).

### 2.12. Statistical Analysis 

Data were presented as mean ± SD (standard deviation). After the normality test, data were analyzed by one-way analysis of variance (ANOVA) followed by multiple comparisons between groups using Tukey’s post hoc test. A Kruskal–Wallis test was used for Garcia scores, beam balance test, and SAH grading scores. The analyses were performed using SPSS version 24.0 (IBM Corp., Armonk, NY, USA). A *p*-value less than 0.05 was considered statistically significant.

## 3. Results

### 3.1. Mortality and SAH Grades

**Figure 2:**
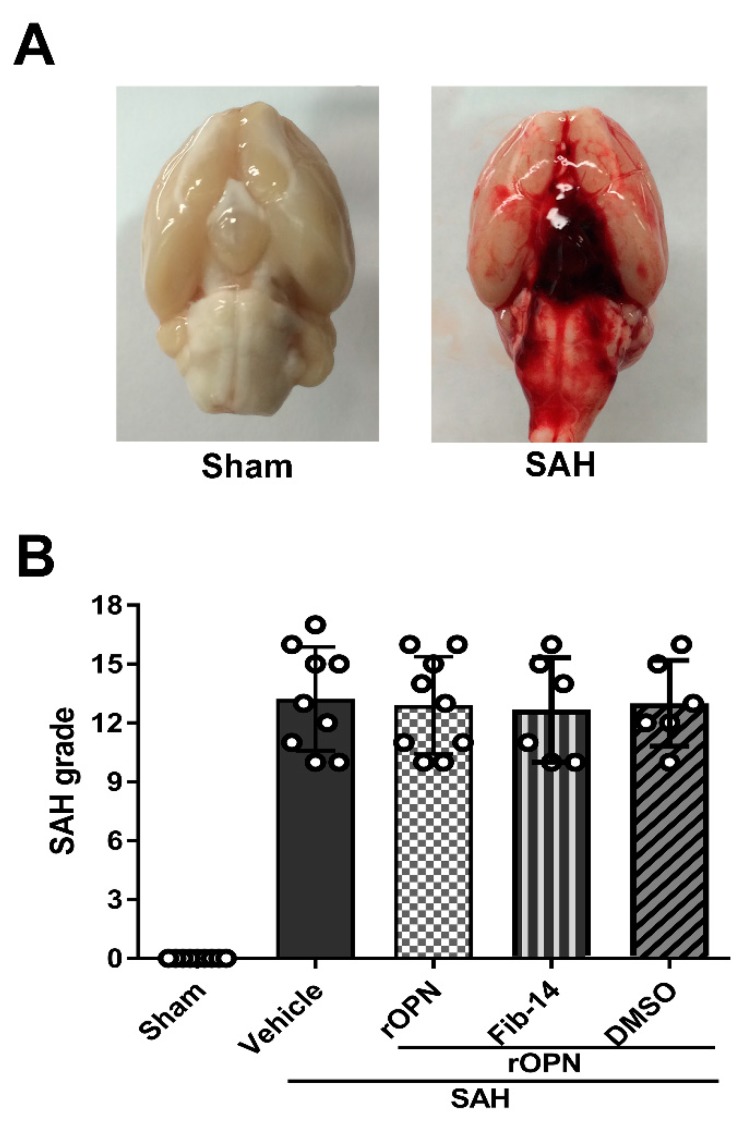
Analysis of subarachnoid hemorrhage (SAH) grades in all SAH groups. (**A**) Representative brain images of Sham and SAH rats. (**B**) Summary of SAH grading scores of all groups. SAH, subarachnoid hemorrhage; rOPN, recombinant OPN; Fib-14, FAK inhibitor 14; DMSO, dimethyl sulfoxide.

A total of 87 rats were used: 68 underwent SAH induction, 19 rats received sham surgery. Five rats were excluded from the study due to mild SAH (SAH grade ≤ 8). The mortality (calculated after exclusion of low-grade rats) of all SAH rats was 20.63% (13/63). No rat died in the Sham group (Table 1). After SAH, blood clots were mainly seen around the circle of Willis and ventral brain stem (Figure 2A). The average SAH grades showed no significant differences among all SAH groups (Figure 2B).

### 3.2. Nasal Administration of rOPN Increased the Expression of Beclin 1 and LC3 in Neurons 24 h after SAH

**Figure 3:**
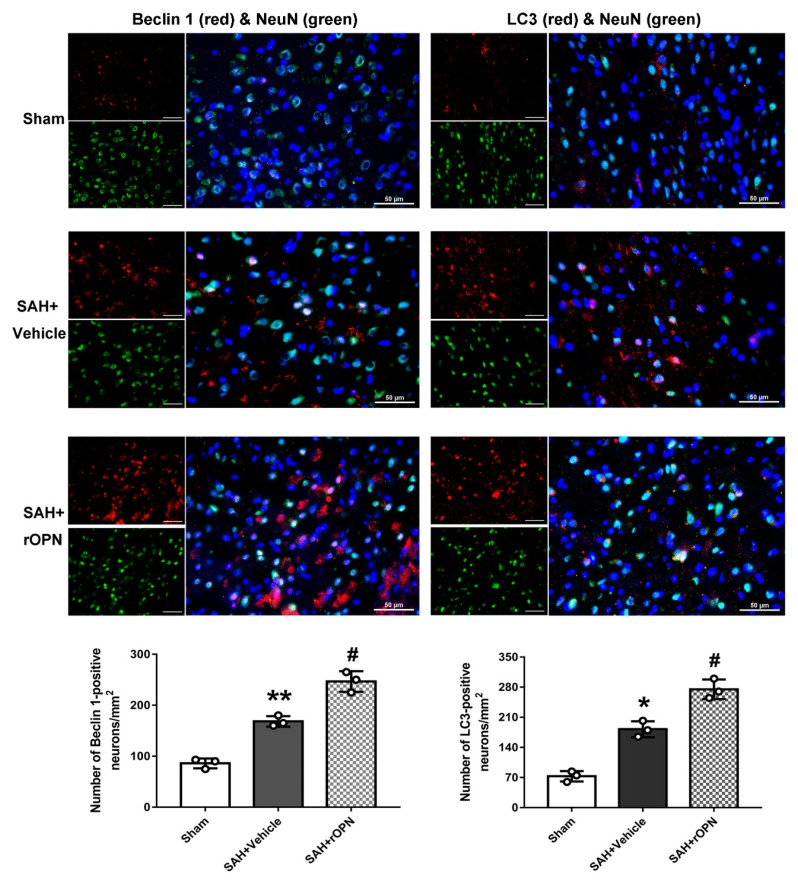
Nasal administration of rOPN increases the expression of Beclin 1 (red fluorescence) and LC3 (red fluorescence) in neurons (green fluorescence). Cell nuclei were counterstained with DAPI (blue fluorescence). *n* = 3 per group. Scale bar = 50 µm. Data are presented as mean ± SD. * *p* < 0.05, ** *p* < 0.01 vs. Sham group; ^#^ *p* < 0.05, vs. SAH + Vehicle group.

Double immunofluorescence was carried out and cells labeling NeuN and Beclin 1 or NeuN and LC3 were counted and compared among the groups. This was done in order to determine whether rOPN administration increases autophagy level in neurons during the early brain injury phase after SAH. Results showed that there were significant increases of Beclin 1-positive neurons and LC3-positive neurons in the SAH + Vehicle group when compared with the Sham group. Moreover, these positive cells significantly increased after rOPN administration in SAH + rOPN group when compared with the Sham group or the SAH + Vehicle group (*p* < 0.05, Figure 3). Thus, indicating that rOPN administration could enhance autophagy in neurons after SAH.

### 3.3. Administration of FAK Inhibitor Abolished Neurobehavioral Improvement Induced by rOPN 

**Figure 4:**
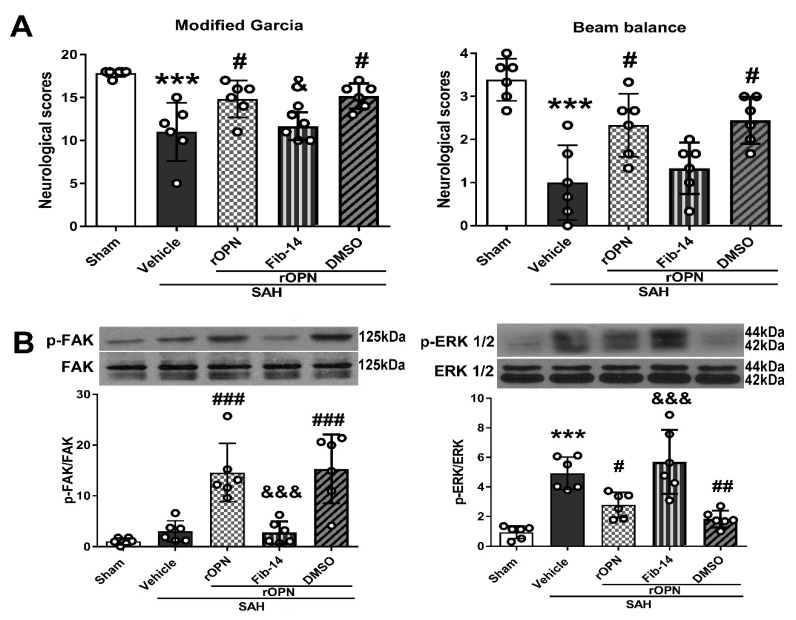
Administration of FAK inhibitor 14 (Fib-14) abolishes neurobehavioral improvement induced by recombinant osteopontin (rOPN) and causes expression changes of downstream protein p-ERK. (**A**) Administration of Fib-14 partially reversed neurobehavioral dysfunction improvement induced by rOPN in modified Garcia and beam balance tests at 24 h after SAH. (**B**) Fib-14 administration significantly inhibited the expression of p-FAK protein while increasing the level of p-ERK 1/2. *n* = 6 per group. Data are presented as mean ± SD. *** *p* < 0.001 vs. Sham group; ^#^ *p* < 0.05, ^##^ *p* < 0.01, ^###^ *p* < 0.001 vs. SAH + Vehicle group; ^&^ *p* < 0.05, ^&&&^ *p* < 0.001 vs. SAH + rOPN + DMSO group. DMSO, dimethyl sulfoxide.

Intranasal administration of rOPN significantly improved neurological scores and increased phosphorylation of FAK 24 h after SAH, compared with SAH rats given vehicle only. Meanwhile, there was a significant decreased phosphorylation level of ERK1/2 in the SAH + rOPN group as compared with the SAH + Vehicle group (*p* < 0.05, Figure 4).

However, pre-SAH intraperitoneal injection of FAK inhibitor-14 (Fib-14) significantly reversed the protective effects of rOPN administration in the SAH + rOPN + Fib-14 group compared with the SAH + rOPN + DMSO group at 24 h after SAH (*p* < 0.05, Figure 4A). Moreover, Fib-14 administration remarkedly decreased the protein level of phosphorylated FAK while the level of phosphorylated ERK1/2 increased significantly (*p* < 0.001, Figure 4B).

### 3.4. FAK Inhibitor Partially Reversed rOPN-Enhanced Autophagy Level in the Brain and Blocked Autophagy Flux after SAH

**Figure 5:**
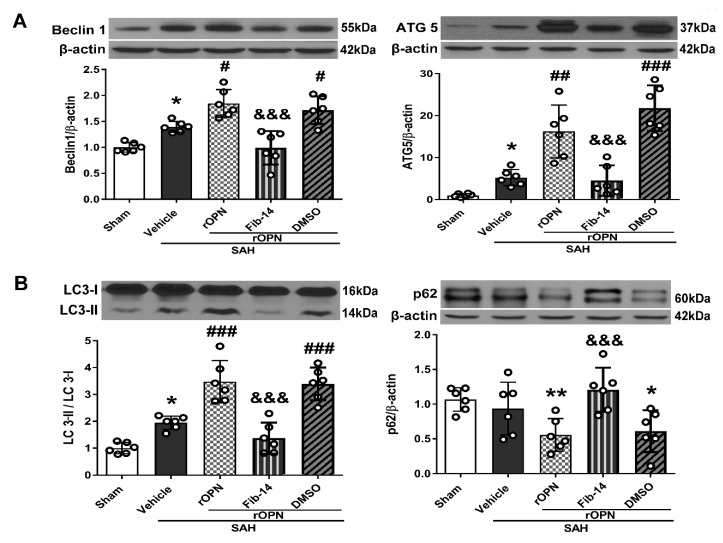
Inhibiting FAK phosphorylation before SAH induction partially reverses recombinant osteopontin (rOPN)-enhanced autophagy and blocks autophagy flux 24 h after SAH. (**A**) FAK inhibitor 14 (Fib-14) administration decreased autophagy level enhanced by rOPN after SAH. (**B**) Fib-14 blocked autophagy flux enhanced by rOPN after SAH. *n* = 6 per group. Data are presented as mean ± SD. * *p* < 0.05, ** *p* < 0.01 vs. Sham group; ^#^ *p* < 0.05, ^##^ *p* < 0.01, ^###^ *p* < 0.001 vs. SAH + Vehicle group; ^&&&^ *p* < 0.001 vs. SAH + rOPN + DMSO group. DMSO, dimethyl sulfoxide.

At 24 h after SAH, expression of autophagy-related proteins (Beclin 1 and ATG 5) were significantly increased in the SAH + Vehicle group compared with the Sham group, and rOPN administration further increased the expression of those proteins in the SAH + rOPN group compared with the SAH + Vehicle group. With the pre-SAH intraperitoneal injection of FAK inhibitor-14 (Fib-14), expression of autophagy-related proteins (Beclin 1 and ATG 5) in the SAH + rOPN + Fib-14 group decreased, as compared with that in the SAH + rOPN + DMSO group, indicating autophagy suppression by Fib-14 administration (*p* < 0.001, Figure 5A). Furthermore, rOPN administration significantly increased LC3 II to I ratio and decreased expression of p62 in the SAH + rOPN group compared with the SAH + Vehicle and Sham groups, respectively. However, conversely, the significantly decreased LC3 II to I ratio and elevated p62 protein level after Fib-14 administration indicated blocked autophagy flux in the SAH + rOPN + Fib-14 group as compared with the SAH + rOPN + DMSO group (*p* < 0.05, Figure 5B).

### 3.5. An Early Nasal Administration of rOPN Improved Long-Term Neurobehavior after SAH 

**Figure 6:**
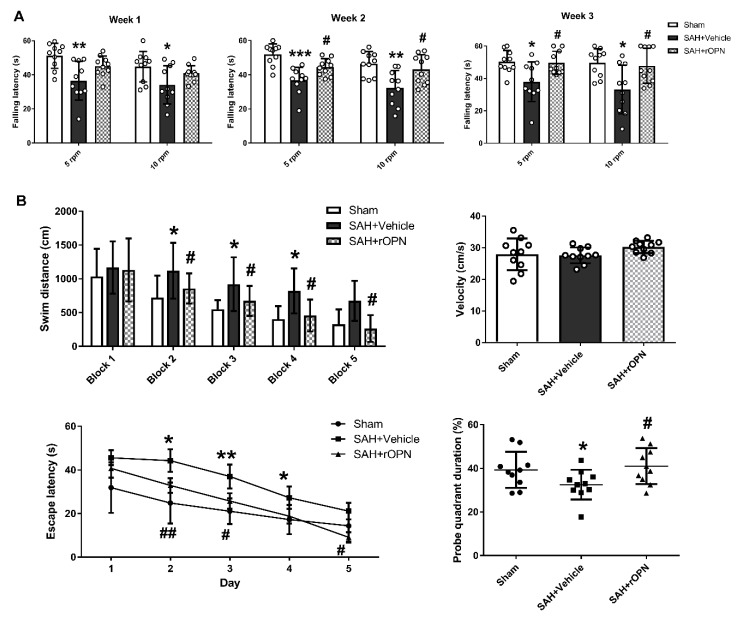
The effects of recombinant osteopontin (rOPN) administration on long-term neurological scores after SAH. (**A**) rOPN increased falling latency in the rotarod test by the end of second and third week after SAH. (**B**) rOPN improved water maze performance on day 22 to 26 after SAH. *n* = 10 per group. Data are presented as mean ± SD. * *p* < 0.05, ** *p* < 0.01, *** *p* < 0.001 vs. Sham group; ^#^ *p* < 0.05, ^##^ *p* < 0.01 vs. SAH + Vehicle group. Block, daily experimental trial.

In the rotarod test by the end of the first, second, and third week after SAH, the SAH + Vehicle group had significantly shorter falling latency in both 5 RPM and 10 RPM tests when compared with Sham. However, falling latency in the SAH + rOPN group was significantly improved by the second and third week as compared with the SAH + Vehicle group (*p* < 0.05, Figure 6A).

In the Morris water maze test, the swimming distance and escape latency increased significantly in the SAH + Vehicle group as compared with the Sham group (*p* < 0.05, Figure 6B). However, both parameters were improved after rOPN administration as indicated by significantly decreased swimming distance since day 2 and significantly reduced escape latency since block 2 (*p* < 0.05, Figure 6B). Swimming velocity throughout the trials was of no significant difference among the three groups, indicating the differences observed in escape latency were not due to different swimming speed. In probe quadrant trials, rats in the SAH + Vehicle group also spent remarkably less time in the target quadrants when compared with the Sham group. rOPN administration also increased the time spent in the target quadrants in the SAH + rOPN group as compared with that in the SAH + Vehicle group (*p* < 0.05, Figure 6B).

### 3.6. Nasal Administration of rOPN Decreased Neuronal Deaths in the Hippocampus Region and Attenuated Delayed Brain Injury after SAH

To investigate the underlying mechanisms for the water maze results, Nissl staining and Fluoro-Jade C staining of the hippocampus were carried out on day 28 after SAH.

**Figure 7:**
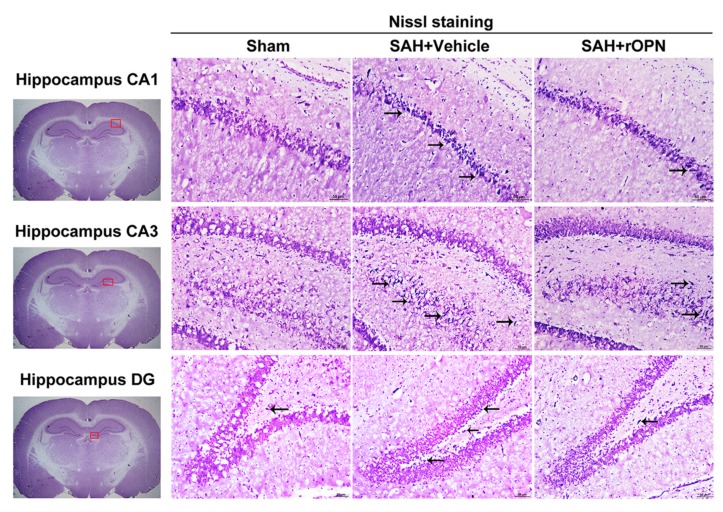
The effects of recombinant osteopontin (rOPN) administration on long-term Nissl staining results after SAH. rOPN decreased the amount of degenerative neurons and attenuated hippocampal injury in the CA1, CA3, and DG sectors in long-term SAH + rOPN rats. *n* = 3 per group. The red boxes in the brain slices images (left side) indicate the locations observed. Arrows indicate degenerative neurons with shrunken cytoplasm and condensed staining. Scale bar = 50 μm. DG, dentate gyrus.

In Nissl staining, the morphology of neurons in the hippocampal tissue was normal in the Sham group. After SAH, there were severe degenerative changes (shrunken cytoplasm and condensed staining) observed in the CA1, CA3, and dentate gyrus sectors. However, these degenerative changes were alleviated in the SAH + rOPN group (Figure 7).

**Figure 8:**
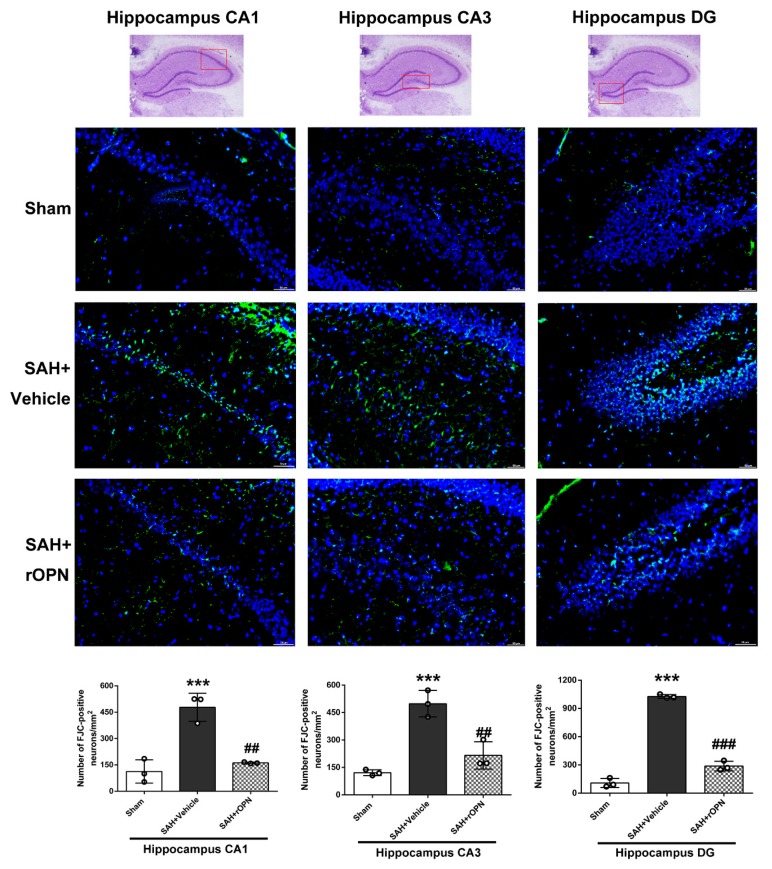
The effects of rOPN administration on long-term Fluoro-Jade C (FJC) results after SAH. rOPN significantly decreased the number of FJC-positive neurons and attenuated hippocampal injury in the CA1, CA3, and DG sectors in long-term SAH + rOPN rats. *n* = 3 per group. Data are presented as mean ± SD. *** *p* < 0.001 vs. Sham group; ^##^ *p* < 0.01^, ###^ *p* < 0.001 vs. SAH + Vehicle group. The red boxes in the hippocampus images on top indicate the locations observed. Scale bar = 50 μm. rOPN, recombinant osteopontin; DG, dentate gyrus.

Fluoro-Jade C staining and consecutive cell count and statistical analysis also revealed a significantly increased number of FJC-positive cells in the SAH + Vehicle group compared with the Sham group in the CA1, CA3, and dentate gyrus sectors (*p* < 0.001, Figure 8). However, the number of FJC-positives cells in all three sectors was significantly decreased in the SAH + rOPN group compared with the SAH + Vehicle group (*p* < 0.01, Figure 8), indicating attenuation of neuronal injury or degeneration by nasal administration of rOPN.

## 4. Discussion

**Figure 9:**
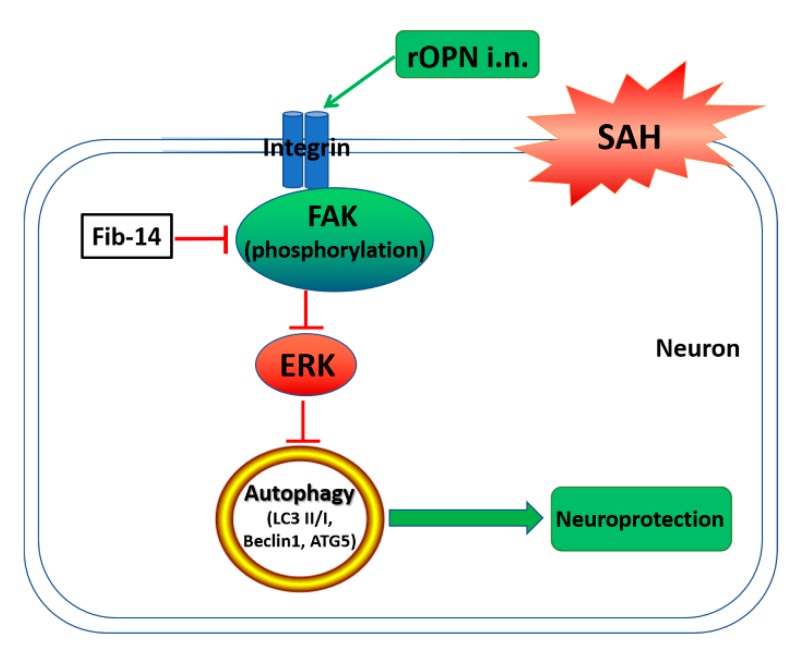
Graphical abstract. SAH, subarachnoid hemorrhage; rOPN, recombinant osteopontin; i.n., intranasal administration; Fib-14, FAK inhibitor 14.

The management of early brain injury (EBI) is a major goal in the treatment of patients with SAH [34]. In recent years, there has been more focus on the role of autophagy and its underlying mechanisms during EBI [13]. Thus, we investigated the effect of osteopontin on autophagy regulation and found that rOPN administration could activate autophagy in neurons 24 h after SAH, probably by regulating the FAK–ERK pathway (Figure 9). Furthermore, we found that early administration of rOPN may be a preventive and therapeutic strategy against delayed brain injury after SAH.

OPN is a neuroprotective glycoprotein expressed in various types of cells. Our previous studies showed that endogenous OPN expression increased after SAH induction [21,35]. Moreover, exogenous OPN treatment showed protective effects against vasospasm and blood-brain barrier disruption after SAH through integrin receptor-dependent activation of MKP-1 and MAPK phosphatase in astrocytes and endothelial cells [18,36]. In experimental SAH models, intranasal administration of rOPN also decreases neuronal apoptosis through FAK/PI3K/Akt-induced inhibition of Caspase-3 cleavage [7]. Regarding the potential effect of OPN on autophagy, one recent study suggested that OPN enhances autophagy via binding with integrin αvβ3 receptor to promote chemo-resistance and cancer stem cell-like phenotype of human hepatocellular carcinoma cells (HCC) [20]. Another study showed that osteopontin stimulates autophagy in vascular smooth muscle cells (VSMC) via both of its receptors: Integrin and CD44 [19]. Our recent previous study further demonstrated the effect of rOPN nasal administration on enhancing autophagy, alleviating apoptosis, and regulating autophagy–apoptosis interaction in a rat model of SAH [21]. In the current study, using the double immunofluorescence method, we determined on the histological level that nasal administration of rOPN increased the expression of Beclin 1 and LC3 in neurons. Our previous study [21] used western blotting to measure the change of the overall amount of autophagy proteins in the whole left hemispheres of rats after SAH. The histological results in the current study provided more evidence that rOPN administration has a direct influence on the autophagy level in neurons at 24 h after SAH.

Previous research indicated that “self-eating” autophagy emerges to be beneficial and can inhibit “self-killing” apoptosis in EBI after experimental SAH [10]. Our previous study also demonstrated that rOPN alleviates EBI, possibly by enhancing autophagy and regulating autophagy–apoptosis interaction [21]. However, there has been a paucity of research regarding the upstream regulation pathway of autophagy after SAH. In the present study, we investigated the possible signaling pathway involved in the rOPN-induced autophagy activation. It has been reported that OPN functions through the binding to its two receptors: Integrin and CD44 [37]. In a previous study by Zheng et al. [19], OPN-induced autophagy was attenuated by blocking the receptor activity using an integrin inhibitor or anti-CD44 antibody. Since focal adhesion kinase (FAK) is one of the most important pathway proteins in the OPN–integrin signaling [22], we investigated in the current study whether blockade of FAK phosphorylation by its specific inhibitor would influence OPN-enhanced autophagy after SAH. We found that after rOPN administration, there was significant increase in the phosphorylation level of FAK, decrease in the phosphorylation level of ERK1/2, and increased autophagy level 24 h after SAH. On the other hand, with the administration of FAK inhibitor, the phosphorylation level of ERK1/2 was drastically elevated, autophagy level in the brain was significantly suppressed, and autophagy flux was also blocked, judging from a significantly elevated p62 level. According to previous studies, ERK is one of the classic downstream proteins to FAK [38,39]. In one of our previous studies, blocking endogenous OPN expression after SAH led to a significant increase in phospho-ERK1/2 level and exacerbated neurologic impairment and BBB disruption [36]. Regarding the role of ERK phosphorylation in autophagy regulation, even though previous results are complicated and controversial conclusions remain, there are evidences suggesting that some agents such as aplasia Ras homolog member I (ARHI; DIRAS3) [40] and pterostilbene [41] can induce autophagy via inhibiting ERK or its upstream molecules as Ras and Map. On the other hand, using specific inhibitors of the Ras–Raf–MEK–ERK axis activates autophagy in human breast cancer cells and human hepatocellular carcinoma (HepG2) cells [42,43]. Combining these previous findings with our results in the current study, we proposed that ERK1/2 phosphorylation may have participated in the OPN–FAK-enhanced autophagy.

Lately, more emphasis has been placed on the investigation of long-term outcomes after SAH [1,4,44], yet previous studies have only focused on short-term neuroprotection of OPN after SAH [7,23,36]. In the current study, we performed a long-term study and found that rOPN administration significantly improved animals’ long-term neurological scores in the rotarod and water maze test. We further observed hippocampal neurons in long-term brain samples and found that SAH caused severe damage and degeneration in CA1, CA3, and dentate gyrus sectors, yet early rOPN administration attenuated these injuries. The injured sectors observed in our current study were consistent with previous studies investigating SAH-induced hippocampal injury [45,46]. Previous studies demonstrated that hippocampal injury began in the EBI phase [47], thus we speculate that the improvement of our single rOPN administration during the EBI phase on long-term outcome might be due to its attenuation of EBI. This is consistent with the proposal by previous research that from a basic research point of view, admission poor clinical grade indicates that severe early brain injury (EBI) is arising at admission and possibly leads to poor outcomes directly or associated with delayed cerebral ischemia [48].

This study has several limitations. First, since previous studies mainly focus on the role of autophagy during the EBI phase, our current study also evaluated the autophagy level in neurons and investigated the possible underlying mechanism at the 24 h time point after SAH. However, the potential effect of autophagy in delayed brain injury after SAH should also be considered and investigated in future experiments. Second, FAK plays multiple roles as one of the most important pathway proteins in the OPN–integrin signaling. Thus, while our western blotting results suggested that the FAK–ERK pathway may be involved in rOPN-enhanced autophagy, we cannot exclude the possibility that blockade of FAK by its inhibitor may also have exerted other effects that caused neurobehavior deterioration. Moreover, investigation about a more detailed signaling pathway such as the role of the Ras/Raf/MER/ERK axis in OPN-enhanced autophagy and its possible connection with upstream pathway proteins such as FAK should be carried out in future studies. In addition, the underlying mechanisms and a more specific time window in the long-term study should be investigated in future studies.

## 5. Conclusions

Our results suggested that rOPN administration in rat models could activate autophagy in neurons 24 h after SAH, and FAK–ERK signaling might be the upstream regulation pathway involved. Furthermore, early administration of rOPN may be a preventive and therapeutic strategy against delayed hippocampal injury after SAH due to its attenuation of early brain injury.

## Figures and Tables

**Table 1:** Summary of animal usage and mortality.

Groups	Mortality	Exclusion
Experiment 1 Short-term study		
Sham	0 (0/3)	0
SAH + Vehicle (PBS)	25% (1/4)	1
SAH + rOPN (5 µg/rat)	25% (1/4)	0
Experiment 2 Mechanism study		
Sham *	0 (0/6)	0
SAH + Vehicle (PBS) *	14.29 (1/7)	2
SAH + rOPN (5 µg/rat) *	25% (2/8)	0
SAH + rOPN + Fib-14	25% (2/8)	1
SAH + rOPN + DMSO	14.29% (1/7)	1
Experiment 3 Long-term study		
Sham	0 (0/10)	0
SAH + Vehicle (PBS)	23.08% (3/13)	0
SAH + rOPN (5 µg/rat)	16.67% (2/12)	0
Total		
Sham	0 (0/19)	0
SAH	20.63% (13/63)	5

SAH: Subarachnoid hemorrhage; PBS: Phosphate-buffered saline; rOPN: Recombinant osteopontin; Fib-14: FAK inhibitor 14; DMSO: Dimethyl sulfoxide; * shared samples with study in reference [21].

## References

[1] Zhu Q., Enkhjargal B., Huang L., Zhang T., Sun C., Xie Z., Wu P., Mo J., Tang J., Xie Z. (2018). Aggf1 attenuates neuroinflammation and BBB disruption via PI3K/Akt/NF-kappaB pathway after subarachnoid hemorrhage in rats. J. Neuroinflammation.

[2] Adil S.M., Liu B., Charalambous L.T., Kiyani M., Gramer R., Swisher C.B., Verbick L.Z., McCabe A., Parente B.A., Pagadala P. (2019). Healthcare Economics of Hydrocephalus After Aneurysmal Subarachnoid Hemorrhage in the United States. Transl. Stroke Res..

[3] Cahill J., Calvert J.W., Zhang J.H. (2006). Mechanisms of early brain injury after subarachnoid hemorrhage. J. Cereb. Blood Flow Metab..

[4] Mo J., Enkhjargal B., Travis Z.D., Zhou K., Wu P., Zhang G., Zhu Q., Zhang T., Peng J., Xu W. (2019). AVE 0991 attenuates oxidative stress and neuronal apoptosis via Mas/PKA/CREB/UCP-2 pathway after subarachnoid hemorrhage in rats. Redox Biol..

[5] Li J.R., Xu H.Z., Nie S., Peng Y.C., Fan L.F., Wang Z.J., Wu C., Yan F., Chen J.Y., Gu C. (2017). Fluoxetine-enhanced autophagy ameliorates early brain injury via inhibition of NLRP3 inflammasome activation following subrachnoid hemorrhage in rats. J. Neuroinflammation.

[6] Chen S., Mei S., Luo Y., Wu H., Zhang J., Zhu J. (2018). Gasdermin Family: A Promising Therapeutic Target for Stroke. Transl. Stroke Res..

[7] Topkoru B.C., Altay O., Duris K., Krafft P.R., Yan J., Zhang J.H. (2013). Nasal administration of recombinant osteopontin attenuates early brain injury after subarachnoid hemorrhage. Stroke.

[8] Yu L., Chen Y., Tooze S.A. (2018). Autophagy pathway: Cellular and molecular mechanisms. Autophagy.

[9] Jing C.H., Wang L., Liu P.P., Wu C., Ruan D., Chen G. (2012). Autophagy activation is associated with neuroprotection against apoptosis via a mitochondrial pathway in a rat model of subarachnoid hemorrhage. Neuroscience.

[10] Wang Z., Shi X.Y., Yin J., Zuo G., Zhang J., Chen G. (2012). Role of autophagy in early brain injury after experimental subarachnoid hemorrhage. J. Mol. Neurosci..

[11] Chen J., Wang L., Wu C., Hu Q., Gu C., Yan F., Li J., Yan W., Chen G. (2014). Melatonin-enhanced autophagy protects against neural apoptosis via a mitochondrial pathway in early brain injury following a subarachnoid hemorrhage. J. Pineal Res..

[12] Meng N., Mu X., Lv X., Wang L., Li N., Gong Y. (2019). Autophagy represses fascaplysin-induced apoptosis and angiogenesis inhibition via ROS and p8 in vascular endothelia cells. Biomed. Pharmacother..

[13] Galluzzi L., Bravo-San Pedro J.M., Blomgren K., Kroemer G. (2016). Autophagy in acute brain injury. Nat. Rev. Neurosci..

[14] Shao A., Wang Z., Wu H., Dong X., Li Y., Tu S., Tang J., Zhao M., Zhang J., Hong Y. (2016). Enhancement of Autophagy by Histone Deacetylase Inhibitor Trichostatin A Ameliorates Neuronal Apoptosis After Subarachnoid Hemorrhage in Rats. Mol. Neurobiol..

[15] Kaleta B., Krata N., Zagozdzon R., Mucha K. (2019). Osteopontin Gene Polymorphism and Urinary OPN Excretion in Patients with Immunoglobulin A Nephropathy. Cells.

[16] Denhardt D.T., Guo X. (1993). Osteopontin: A protein with diverse functions. FASEB J..

[17] Fisher L.W., Torchia D.A., Fohr B., Young M.F., Fedarko N.S. (2001). Flexible structures of SIBLING proteins, bone sialoprotein, and osteopontin. Biochem. Biophys. Res. Commun..

[18] Suzuki H., Hasegawa Y., Chen W., Kanamaru K., Zhang J.H. (2010). Recombinant osteopontin in cerebral vasospasm after subarachnoid hemorrhage. Ann. Neurol..

[19] Zheng Y.H., Tian C., Meng Y., Qin Y.W., Du Y.H., Du J., Li H.H. (2012). Osteopontin stimulates autophagy via integrin/CD44 and p38 MAPK signaling pathways in vascular smooth muscle cells. J. Cell Physiol..

[20] Liu G., Fan X., Tang M., Chen R., Wang H., Jia R., Zhou X., Jing W., Wang H., Yang Y. (2016). Osteopontin induces autophagy to promote chemo-resistance in human hepatocellular carcinoma cells. Cancer Lett..

[21] Sun C., Enkhjargal B., Reis C., Zhou K., Xie Z., Wu L., Zhang T., Zhu Q., Tang J., Jiang X. Osteopontin attenuates early brain injury through regulating autophagy-apoptosis interaction after subarachnoid hemorrhage in rats. CNS Neurosci. Ther..

[22] Visavadiya N.P., Keasey M.P., Razskazovskiy V., Banerjee K., Jia C., Lovins C., Wright G.L., Hagg T. (2016). Integrin-FAK signaling rapidly and potently promotes mitochondrial function through STAT3. Cell Commun. Signal..

[23] Wu J., Zhang Y., Yang P., Enkhjargal B., Manaenko A., Tang J., Pearce W.J., Hartman R., Obenaus A., Chen G. (2016). Recombinant Osteopontin Stabilizes Smooth Muscle Cell Phenotype via Integrin Receptor/Integrin-Linked Kinase/Rac-1 Pathway After Subarachnoid Hemorrhage in Rats. Stroke.

[24] Gong L., Manaenko A., Fan R., Huang L., Enkhjargal B., McBride D., Ding Y., Tang J., Xiao X., Zhang J.H. (2018). Osteopontin attenuates inflammation via JAK2/STAT1 pathway in hyperglycemic rats after intracerebral hemorrhage. Neuropharmacology.

[25] Peng J., Pang J., Huang L., Enkhjargal B., Zhang T., Mo J., Wu P., Xu W., Zuo Y., Peng J. (2019). LRP1 activation attenuates white matter injury by modulating microglial polarization through Shc1/PI3K/Akt pathway after subarachnoid hemorrhage in rats. Redox Biol..

[26] Xie Z., Enkhjargal B., Reis C., Huang L., Wan W., Tang J., Cheng Y., Zhang J.H. (2017). Netrin-1 Preserves Blood-Brain Barrier Integrity Through Deleted in Colorectal Cancer/Focal Adhesion Kinase/RhoA Signaling Pathway Following Subarachnoid Hemorrhage in Rats. J. Am. Heart Assoc..

[27] Garcia J.H., Wagner S., Liu K.-F., Hu X.-j. (1995). Neurological Deficit and Extent of Neuronal Necrosis Attributable to Middle Cerebral Artery Occlusion in Rats. Stroke.

[28] Chen H., Burris M., Fajilan A., Spagnoli F., Tang J., Zhang J.H. (2011). Prolonged Exposure to Isoflurane Ameliorates Infarction Severity in the Rat Pup Model of Neonatal Hypoxia-Ischemia. Transl. Stroke Res..

[29] Sherchan P., Lekic T., Suzuki H., Hasegawa Y., Rolland W., Duris K., Zhan Y., Tang J., Zhang J.H. (2011). Minocycline improves functional outcomes, memory deficits, and histopathology after endovascular perforation-induced subarachnoid hemorrhage in rats. J. Neurotrauma.

[30] Sozen T., Tsuchiyama R., Hasegawa Y., Suzuki H., Jadhav V., Nishizawa S., Zhang J.H. (2009). Role of Interleukin-1β in Early Brain Injury After Subarachnoid Hemorrhage in Mice. Stroke.

[31] Li L., Tao Y., Tang J., Chen Q., Yang Y., Feng Z., Chen Y., Yang L., Yang Y., Zhu G. (2015). A Cannabinoid Receptor 2 Agonist Prevents Thrombin-Induced Blood-Brain Barrier Damage via the Inhibition of Microglial Activation and Matrix Metalloproteinase Expression in Rats. Transl. Stroke Res..

[32] Gittins R., Harrison P.J. (2004). Neuronal density, size and shape in the human anterior cingulate cortex: A comparison of Nissl and NeuN staining. Brain Res. Bull..

[33] Shi X., Xu L., Doycheva D.M., Tang J., Yan M., Zhang J.H. (2016). Sestrin2, as a negative feedback regulator of mTOR, provides neuroprotection by activation AMPK phosphorylation in neonatal hypoxic-ischemic encephalopathy in rat pups. J. Cereb. Blood Flow Metab..

[34] Neulen A., Meyer S., Kramer A., Pantel T., Kosterhon M., Kunzelmann S., Goetz H., Thal S.C. (2018). Large Vessel Vasospasm Is Not Associated with Cerebral Cortical Hypoperfusion in a Murine Model of Subarachnoid Hemorrhage. Transl. Stroke Res..

[35] Enkhjargal B., McBride D.W., Manaenko A., Reis C., Sakai Y., Tang J., Zhang J.H. (2017). Intranasal administration of vitamin D attenuates blood-brain barrier disruption through endogenous upregulation of osteopontin and activation of CD44/P-gp glycosylation signaling after subarachnoid hemorrhage in rats. J. Cereb. Blood Flow Metab..

[36] Suzuki H., Hasegawa Y., Kanamaru K., Zhang J.H. (2010). Mechanisms of osteopontin-induced stabilization of blood-brain barrier disruption after subarachnoid hemorrhage in rats. Stroke.

[37] Agnihotri R., Crawford H.C., Haro H., Matrisian L.M., Havrda M.C., Liaw L. (2001). Osteopontin, a Novel Substrate for Matrix Metalloproteinase-3 (Stromelysin-1) and Matrix Metalloproteinase-7 (Matrilysin). J. Biol. Chem..

[38] Ku M.J., Kim J.H., Lee J., Cho J.Y., Chun T., Lee S.Y. (2015). Maclurin suppresses migration and invasion of human non-small-cell lung cancer cells via anti-oxidative activity and inhibition of the Src/FAK–ERK–β-catenin pathway. Mol. Cell. Biochem..

[39] Viale-Bouroncle S., Gosau M., Morsczeck C. (2014). Laminin regulates the osteogenic differentiation of dental follicle cells via integrin-α2/-β1 and the activation of the FAK/ERK signaling pathway. Cell Tissue Res..

[40] Lu Z., Yang H., Sutton M.N., Yang M., Clarke C.H., Liao W.S., Bast R.C. (2014). ARHI (DIRAS3) induces autophagy in ovarian cancer cells by downregulating the epidermal growth factor receptor, inhibiting PI3K and Ras/MAP signaling and activating the FOXo3a-mediated induction of Rab7. Cell Death Differ..

[41] Ko C.P., Lin C.W., Chen M.K., Yang S.F., Chiou H.L., Hsieh M.J. (2015). Pterostilbene induce autophagy on human oral cancer cells through modulation of Akt and mitogen-activated protein kinase pathway. Oral Oncol..

[42] Ouyang L., Chen Y., Wang X.-y., Lu R.-f., Zhang S.-y., Tian M., Xie T., Liu B., He G. (2014). Polygonatum odoratum lectin induces apoptosis and autophagy via targeting EGFR-mediated Ras-Raf-MEK-ERK pathway in human MCF-7 breast cancer cells. Phytomedicine.

[43] Zhao Y., Chen H., Shang Z., Jiao B., Yuan B., Sun W., Wang B., Miao M., Huang C. (2012). SD118-xanthocillin X (1), a novel marine agent extracted from Penicillium commune, induces autophagy through the inhibition of the MEK/ERK pathway. Mar. Drugs.

[44] Zhang T., Wu P., Budbazar E., Zhu Q., Sun C., Mo J., Peng J., Gospodarev V., Tang J., Shi H. (2019). Mitophagy Reduces Oxidative Stress Via Keap1 (Kelch-Like Epichlorohydrin-Associated Protein 1)/Nrf2 (Nuclear Factor-E2-Related Factor 2)/PHB2 (Prohibitin 2) Pathway After Subarachnoid Hemorrhage in Rats. Stroke.

[45] Jeon H., Ai J., Sabri M., Tariq A., Macdonald R.L. (2010). Learning deficits after experimental subarachnoid hemorrhage in rats. Neuroscience.

[46] Dong Y., Li Y., Feng D., Wang J., Wen H., Liu D., Zhao D., Liu H., Gao G., Yin Z. (2013). Protective effect of HIF-1alpha against hippocampal apoptosis and cognitive dysfunction in an experimental rat model of subarachnoid hemorrhage. Brain Res..

[47] He Z., Ostrowski R.P., Sun X., Ma Q., Huang B., Zhan Y., Zhang J.H. (2012). CHOP silencing reduces acute brain injury in the rat model of subarachnoid hemorrhage. Stroke.

[48] Suzuki H. (2019). Inflammation: A Good Research Target to Improve Outcomes of Poor-Grade Subarachnoid Hemorrhage. Transl. Stroke Res..

